# Body composition from 18 to 22 years and pulmonary function at 22 years—1993 Pelotas Birth Cohort

**DOI:** 10.1371/journal.pone.0219077

**Published:** 2019-06-27

**Authors:** Paula Duarte Oliveira, Fernando C. Wehrmeister, Helen Gonçalves, Maria Cecília Assunção, Rogelio Peréz-Padilla, Fernando C. Barros, Ana Maria Baptista Menezes

**Affiliations:** 1 Federal University of Pelotas, Postgraduate Program in Epidemiology, Pelotas, Brazil; 2 Instituto Nacional de Enfermedades Respiratorias, Institute of Respiratory Diseases, Mexico City, Mexico; 3 Catholic University of Pelotas, Postgraduate Program in Health and Behavior, Pelotas, Brazil; Taipei City Hospital, TAIWAN

## Abstract

The objective was to verify the association between body composition from 18 to 22 years and pulmonary function at 22 years of age. This longitudinal analysis was conducted with a Brazilian birth cohort data. The outcomes were the forced expiratory volume in the first second and forced vital capacity (FVC), measured at 22 years follow-up. Main exposures: obesity (body mass index ≥ 30kg/m^2^), and highest tertiles of fat mass (FM) and fat mass index (air displacement plethysmography) measured at 18 and 22 years-old follow-ups. The reference category (not exposed) was defined by those individuals who were not classified in the highest adiposity categories mentioned, in both ages. Multivariable linear regressions stratified by sex were used. The sample comprised 3,511 participants. Those who belonged to the highest adiposity categories in 18 and 22 years follow-ups showed lower pulmonary function at 22 years when compared to those who were not classified in the higher adiposity categories in both ages (reference category); those in the highest tertile of FM showed a mean FVC -313mL (95%CI -421; -206) and -259mL (95%CI -336; -182) in men and women, compared to the reference category, respectively. Those who changed from the higher to the lower adiposity categories (from 18 to 22 years) showed pulmonary function similar to the reference, and those who presented the opposite body composition trajectory, showed decreased pulmonary function results at 22 years, mainly among women. We concluded that high body adiposity in two follow-ups and especially contemporary adiposity was associated with lower pulmonary function at 22 years.

## Introduction

Overweight/obesity is a risk factor to several heath conditions [1] and it has been associated also to decreased pulmonary function[2]. These findings have been attributed to the restriction and load imposed by excess fat to pulmonary mechanics [3, 4], with adipose tissue restricting both diaphragmatic and ribcage expansion[4]. Systemic inflammation is also pointed out as a possible explanation linking adiposity and pulmonary function, as it can lead to airway inflammation [3, 5].

Many of the previous studies including adolescent and young adults are cross-sectional and assess exposures using anthropometric measures, such as body mass index (BMI) [6–8]. However, anthropometric measures are unable to distinguish fat mass (FM) from fat-free mass [9], with opposite impact on pulmonary function [10, 11]. This leads to conflicting results in the literature, when comparing studies using anthropometric exposures with those using measures able to distinguish body components [3, 12–15].

Studies using accurate methods to evaluate body composition, such as dual energy x-ray absorptiometry (DXA) or air displacement plethysmography (BOD POD), are mostly cross-sectional and target very specific populations [3, 16–18]. Longitudinal studies using these precise methods and their relationship to pulmonary function are rare [19], and are especially important since the rates of overweight and obesity, known as modifiable risk factors, are growing in several countries and impacting on the population health [1].

Therefore, the present study aims to analyze changes in body composition between 18 and 22 years of age in a birth cohort, using, in addition to BMI, high-precision body adiposity measures, and its repercussion on spirometry results at 22 years.

## Methods

This is a longitudinal analysis conducted with a birth cohort data (1993 Birth Cohort, Pelotas, Southern Brazil). In 1993, all live births whose mothers were living in the urban area of Pelotas (about 340,000 inhabitants, 7m above sea level) were considered eligible for the study. Cohort members were followed at several times throughout their lives. For the purposes of this study, we used data from the perinatal, 2011 and 2015 follow-ups, when the participants were 18 and 22 years of age: in both, the participants were invited to attend the clinic (Epidemiology Research Center) for questionnaires and examinations. At 22 years, the instrument for data collection was the Research Electronic Data Capture (REDCap)[20]. Further details on cohort methodology can be found in previous publications [21, 22].

All participants who underwent pulmonary function tests at 22-years follow-up were included in the analyses. The outcomes were pre-bronchodilator forced expiratory volume in the first second (FEV_1_) and forced vital capacity (FVC) expressed in liters and FEV_1_/FVC ratio expressed as percentage, measured by a portable ultrasonic spirometer (Easy One model, nDD Medical Technologies Inc., Zurich, Switzerland). The exclusion criteria for the spirometry, as reported by the participants, were: active tuberculosis, pregnancy, cardiac problems, and thoracic, ocular surgery and retinal displacement in the last three months. All examinations underwent quality control, according to the guidelines of the American Thoracic Society/European Respiratory Society (ATS/ERS) [23]—three acceptable maneuvers with a maximum difference of 150mL between the two highest values for FVC and FEV_1_.

The main exposures were percentage of fat mass (FM—%) and the fat mass index (FMI—total fat mass in kilograms divided by height in m^2^), both measured through air displacement plethysmography (BOD POD Composition System, COSMED, Albano Laziale, Italy) and BMI (kg/m^2^). Total weight was measured using BOD POD scale and height by a stadiometer. These measures were obtained at 18 and 22-years of age. To evaluate body composition changes between such follow-ups, FM and FMI were divided into tertiles, stratified by sex, and the highest tertile (highest adiposity) was considered the exposure category. The variables generated for FM and FMI, combining the two moments of measurement were: never in the highest tertile, only at 18, only at 22 and at the highest tertile in both ages. For the evaluation of BMI changes, the variable was dichotomized at the cut-off point defining obesity (≥ 30kg/m^2^) [24] and participants categorized as never obese, obese only at 18 years of age, only at 22 and obese in both. The reference category comprised non-obese individuals (by BMI), and who were not in the highest tertile of FM and FMI in both ages.

The variables were described as means and standard deviations (SD) for continuous variables and as absolute and relative frequencies for categorical variables. To verify the associations between the changes in FM, FMI and BMI, and pulmonary function at 22 years, multivariable linear regression models were used. There was a modification effect between sex and body fat variables in most of the analyses; therefore, all the analyses were stratified by sex. The covariates, defined a priori and included at the same time in the analysis model, were birthweight and maternal smoking at any time during pregnancy, collected in the perinatal interview; from the 22-years follow-up: height, weight, skin color, complete years of study, household socioeconomic level (asset index), use of any type of corticosteroids in the last three months and physical activity (total minutes/week adding leisure and commuting activities, the minutes of vigorous exercises were multiplied by two); from the 18 and 22-years follow-ups combined covariates were: self-reported wheezing in the last year prior to the follow-up and current smoking (at least one cigarette/week in the last month), both variables classified as no, only at 18, only at 22 year, and both ages. Additionally, an adjusted analysis including pulmonary function at 18 years old was carried out and is available as supplementary material. The analyses were performed in Stata 12.2 (Stata Corp., College Station, Texas, USA) and the values of p<0.05 were considered statistically significant by the Wald test of heterogeneity.

The cohort follow-up projects were approved by the Ethics Committee of the Universidade Federal de Pelotas (protocols 05/11 and 1,250,366, at 18 and 22-years follow-ups, respectively) and the cohort members signed free informed consent form prior to each participation.

## Results

The cohort initial sample comprised 5,249 subjects born in 1993. The follow-up rate at 18 and 22 years was 81.3% and 76.3%, respectively (rates calculated based on the 3,810 answered questionnaires plus 193 deaths). The participants who performed spirometry were 3,511, being 1,832 females. A total of 134 participants met at least one of the exclusion criteria and the main reason was pregnancy (54 women). Also, 14 subjects refused to perform the test. The quality criteria, according to ATS/ERS guidelines (32), were reached in 89.4% of the spirometric tests.

Table 1 describes the study sample distribution and mean FEV_1_ and FVC according to demographic, socioeconomic, behavioral, health characteristics, and the main exposures at 22 years. Most participants were white (about 63% for both sexes) and never smoked (69% and 76%, men and women, respectively). Forty percent of men and 42% of women had between 9 to 11 years of schooling. The exposure variables, FM, FMI and BMI categories were mostly maintained in both evaluations: only 20% changed the adiposity category and belonged to the risk group only at 18 or 22 years of age. Around 80% of the males and ¾ of the females presented a BMI lower than 30 kg/m^2^ in both follow-ups.

**Table 1:** Description of the sample and pulmonary function (individuals who underwent spirometry at age 22) according to demographic, socioeconomic, behavioral, health and body composition changes, stratified by sex.

	Males (n = 1,679)			Females (n = 1,832)		
	N (%)	FEV_1_ (L) Mean (SE)	FVC (L) Mean (SE)	N (%)	FEV_1_ (L) Mean (SE)	FVC (L) Mean (SE)
Birth weight (grams)*		p< 0.001	p< 0.001		p< 0.001	p< 0.001
≥2500	114 (6.8)	3,86 (0.06)	4.64 (0.07)	196 (10.7)	2.82 (0.03)	3.30 (0.04)
<2500	1,564 (93.2)	4.14 (0.02)	4.95 (0.02)	1,635 (89.3)	3.03 (0.01)	3.54 (0.01)
**Maternal smoking during pregnancy**		p = 0.009	p = 0.051		p< 0.001	p = 0.001
No	1,149 (68.4)	4.15 (0.02)	4.95 (0.02)	1,223 (66.8)	3.04 (0.01)	3.54 (0.01)
Yes	530 (31.6)	4.06 (0.03)	4.88 (0.03)	609 (33.2)	2.94 (0.02)	3.46 (0.02)
Skin color*		p< 0.001	p< 0.001		p< 0.001	p< 0.001
White	1,005 (63.7)	4.20 (0.02)	5.03 (0.02)	1,106 (63.0)	3.07 (0.01)	3.58 (0.02)
Black	234 (14.8)	3.65 (0.04)	4.58 (0.05)	266 (15.2)	2.86 (0.03)	3.34 (0.03)
Brown	269 (17.1)	4.07 (0.04)	4.87 (0.04)	323 (18.4)	2.94 (0.03)	3.46 (0.03)
Others	69 (4.4)	4.05(0.08)	4.82 (0.09)	60 (3.4)	3.03 (0.06)	3.54 (0.07)
**Schooling (years)**		p< 0.001	p< 0.001		p< 0.001	p< 0.001
0–4	59 (3.5)	3.71 (0.08)	4.42 (0.09)	30 (1.6)	2.70 (0.08)	3.11 (0.09)
5–8	549 (32.8)	3.99 (0.03)	4.79 (0.03)	387 (21.2)	2.85 (0.02)	3.37 (0.03)
9–11	674 (40.3)	4.17 (0.02)	4.95 (0.03)	770 (42.1)	3.02 (0.02)	3.54 (0.02)
≥ 12	392 (23.4)	4.30 (0.03)	5.16 (0.04)	643 (35.1)	3.09 (0.02)	3.59 (0.02)
**Corticoids use in the last three months**		p = 0.001	p = 0.455		p = 0.186	p = 0.972
No	1,560 (97.7)	4.13 (0.02)	4.92 (0.02)	1,691 (96.4)	3.00 (0.01)	3.51 (0.01)
Yes	36 (2.3)	3.77 (0.11)	4.83 (0.12)	63 (3.6)	2.92 (0.06)	3.51 (0.06)
**Total physical activity ≥ 150 minutes/week** **(leisure and commuting)**		p = 0.841	p = 0.775		p = 0.697	p = 0.129
No	429 (36.1)	4,12 (0.03)	4.94 (0.04)	760 (41.6)	3.01 (0.02)	3.49 (0.02)
Yes	1,249 (74.4)	4.13 (0.02)	4.93 (0.02)	1,068 (58.4)	3.00 (0.01)	3.53 (0.02)
**Current smoker at 18 and 22 years**		p = 0.085	p = 0.438		p = 0.006	p = 0.516
No	1,276 (80.2)	4.13 (0.02)	4.93 (0.02)	1,388 (81.7)	3.03 (0.01)	3.52 (0.01)
Only at 18	14 (0.9)	4.05 (0.17)	4.93 (0.19)	16 (0.9)	2.84 (0.11)	3.36 (0.13)
Only at 22	88 (5.5)	4.20 (0.07)	5.04 (0.08)	109 (6.4)	2.93 (0.04)	3.48 (0.05)
Both ages	213 (13.4)	4.03 (0.04)	4.89 (0.05)	186 (11.0)	2.94 (0.03)	3.52 (0.04)
Wheezing in the last 12 months at 18 and 22 years*		p< 0.001	p = 0.481		p< 0.001	p = 0.132
No	1,303 (82.1)	4.16 (0.02)	4.94 (0.02)	1,372 (80.9)	3.03 (0.01)	3.53 (0.01)
Only at 18	110 (6.9)	4.00 (0.06)	4.85 (0.07)	165 (9.7)	2.95 (0.03)	3.49 (0.04)
Only at 22	109 (6.9)	3.97 (0.06)	4.94 (0.07)	93 (5.5)	2.84 (0.05)	3.41 (0.05)
Both ages	66 (4.2)	3.78 (0.08)	4.84 (0.09)	65 (3.8)	2.92 (0.06)	3.57 (0.06)
**BMI (kg/m**^**2**^**)**		p = 0.083	p< 0.001		p = 0.024	p< 0.001
<18.5	60 (3.6)	3.94 (0.08)	4.51 (0.09)	88 (4.8)	2.89 (0.05)	3.24 (0.05)
18.5–24.9	878 (52.4)	4.14 (0.02)	4.90 (0.02)	934 (51.0)	3.03 (0.01)	3.50 (0.02)
25.0–29.9	503 (30.0)	4.15 (0.03)	5.02 (0.03)	457 (25.0)	3.01 (0.02)	3.56 (0.02)
≥ 30.0	234 (14.0)	4.09 (0.04)	4.96 (0.05)	351 (19.2)	2.97 (0.02)	3.55 (0.03)
**Fat mass (%) in the highest tertile at 18 and 22 years**		p = 0.001	p = 0.004		p = 0.001	p = 0.167
No	917 (58.1)	4.13 (0.02)	4.92 (0.02)	994 (59.0)	3.03 (0.01)	3.52 (0.02)
Only at 18	156 (9.9)	4.30 (0.05)	5.13 (0.06)	141 (8.4)	3.11 (0.04)	3.62 (0.04)
Only at 22	135 (8.6)	4.11 (0.05)	4.93 (0.06)	152 (9.0)	2.95 (0.04)	3.51 (0.04)
Both ages	371 (23.5)	4.06 (0.03)	4.89 (0.04)	399 (23.7)	2.96 (0.02)	3.51 (0.03)
**FMI in the highest tertile at 18 and 22 years**		p< 0.001	p< 0.001		p = 0.002	p = 0.003
No	911 (57.8)	4.11 (0.03)	4.90 (0.02)	1,000 (59.3)	3.01 (0.01)	3.49 (0.02)
Only at 18	139 (8.8)	4.39 (0.05)	5.21 (0.06)	134 (8.0)	3.14 (0.04)	3.66 (0.04)
Only at 22	135 (8.6)	4.10 (0.05)	4.95 (0.06)	143 (8.5)	2.94 (0.04)	3.52 (0.05)
Both ages	392 (24.9)	4.08 (0.03)	4.92 (0.04)	409 (24.3)	2.99 (0.02)	3.54 (0.03)
**BMI ≥30 kg/m**^**2**^ **at 18 and 22 years**		p = 0.161	p = 0.104		p = 0.313	p = 0.557
No	1,337 (80.9)	4.12 (0.02)	4.92 (0.02)	1,353 (76.0)	3.02 (0.01)	3.51 (0.01)
Only at 18	77 (4.7)	4.36 (0.12)	5.25 (0.14)	61 (3.4)	3.01 (0.10)	3.53 (0.11)
Only at 22	136 (8.2)	4.13 (0.05)	4.99 (0.06)	208 (11.7)	2.97 (0.03)	3.53 (0.04)
Both ages	103 (6.2)	4.05 (0.06)	4.93 (0.07)	159 (8.9)	2.97 (0.04)	3.57 (0.04)

FEV1: forced expiratory volume in the first second; FVC: forced vital capacity; All information collected at 18 and/or 22 years follow-up, except birthweight and maternal smoking during gestation, collected in the perinatal follow-up, and skin color, collected at 15-years follow-up. P-value by Wald’s test for heterogeneity.

*Variables missing information (maximum of 228 missings for wheezing).

Body adiposity increased during follow-up (see Table 2) including a rise in mean FM of 4.1% in males and 3.1% in females. On the other hand, there were no significant changes in the mean values of FEV_1_ and FVC between the ages of 18 and 22 years.

**Table 2:** Body composition and pulmonary function at 18 and 22 years old, stratified by sex (individuals who underwent spirometry at age 22).

	Males (n = 1.679) mean (SD)		Females (n = 1.832) mean (SD)	
	18y	22y	18y	22y
**Weight (kg)**	70.7 (14.3)	76.3 (16.1)	60.8 (12.9)	66.1 (15.5)
**Height (cm)**	173.8 (6.9)	174.5 (7.0)	161.1 (6.4)	161.2 (6.6)
**BMI (kg/m**^**2**^**)**	23.3 (4.1)	25.0 (4.8)	23.4 (4.7)	25.4 (5.7)
**Fat mass (%)**	16.7 (8.8)	20.8 (9.8)	32.6 (7.7)	35.7 (8.6)
**FMI (kg/m**^**2**^**)**	4.2 (3.0)	5.6 (3.7)	7.9 (3.5)	9.5 (4.4)
**FEV**_**1**_ **(L)**	4.1 (0.6)	4.1 (0.6)	3.0 (0.4)	3.0 (0.5)
**FVC (L)**	4.8 (0.7)	4.9 (0.7)	3.5 (0.5)	3.5 (0.5)
**FEV**_**1**_**/FVC (%)**	86.0 (6.9)	83.8 (6.8)	87.2 (6.4)	85.7 (6.1)

BMI: body mass index; FMI: fat mass index; FEV_1_: forced expiratory volume in the first second; FVC: forced vital capacity; 1,575 male and 1,646 female with information at 18 years and spirometry at 22-years follow-up.

Associations between pulmonary function and changes in body adiposity category from 18 to 22 years or the maintenance on the highest tertile of FM or FMI or BMI ≥30kg/m^2^ in comparison to those who have never been in these categories (reference) are described in Table 3 (males) and 4 (females). A higher body fat at 18 and 22 years was associated with lower FEV_1_ and FVC at 22 years when compared to the reference category in both sexes (except for FEV_1_ and BMI, among males).

**Table 3:** Adjusted linear regressions between body adiposity from 18 to 22 years and pulmonary function measured at 22 years, males (n = 1494).

	Males - 22y		
	FEV_1_ (L) β (95% CI)	FVC (L) β (95% CI)	FEV_1_/FVC (%) β (95% CI)
**Fat mass (%) in the highest tertile at 18 and 22 years**	p< 0.001	p< 0.001	p = 0.031
No	Reference (0)	Reference (0)	Reference (0)
Only at 18	0.052 (-0.040; 0.145)	0.012 (-0.090; 0.113)	0.792 (-0.373; 1.957)
Only at 22	-0.071 (-0.175; 0.034)	-0.153 (-0.268; -0.038)	1.216 (-0,099; 2.531)
Both ages	-0.244 (-0.336; -0.152)	-0.395 (-0.495; -0.293)	1.686 (0.528; 2.844)
**FMI in the highest tertile at 18 and 22 years**	p< 0.001	p< 0.001	p = 0.112
No	Reference (0)	Reference (0)	Reference (0)
Only at 18	0.146 (0.047; 0.245)	0.100 (-0.008; 0.209)	1.193 (-0.052; 2.439)
Only at 22	-0.060 (-0.168; 0.047)	-0.101 (-0.220; 0.018)	0.649 (-0.708; 2.006)
Both ages	-0.198 (-0.296; -0.101)	-0.313 (-0.421; -0.206)	1.279 (0.052; 2.507)
**BMI ≥30 kg/m**^**2**^ **at 18 and 22 years**	p = 0.160	p = 0.014	p = 0.382
No	Reference (0)	Reference (0)	Reference (0)
Only at 18	0.123 (-0.087; 0.332)	0.108 (-0.124; 0.339)	0.717 (-1.903; 3.336)
Only at 22	-0.016 (-0.138; 0.106)	-0.097 (-0.232; 0.038)	1.035 (-0.489; 2.560)
Both ages	-0.135 (-0.287; 0.017)	-0.256 (-0.426; -0.088)	1.542 (-0.365; 3.449)

BMI: body mass index; FMI: fat mass index; FEV_1_: forced expiratory volume in the first second; FVC: forced vital capacity; β: regression coefficient; p-value by Wald’s test for heterogeneity. ADJUSTED for birth weight and maternal smoking in pregnancy, skin color, self-reported wheezing in the last year at 18 and 22 years, current smoking at 18 and 22 years, and height, weight, physical activity (minutes/week), use of corticosteroids in the last three months, education and asset index at 22 years follow-up.

The changes from the lowest tertiles to the highest FM or FMI tertile or BMI ≥ 30 kg/m^2^ were associated with lower pulmonary function in all analyses for females (except FVC and FMI—Table 4). In males, only the change to the highest tertile of FM at age 22 was associated with lower FVC (Table 3).

**Table 4:** Adjusted linear regressions between body adiposity from 18 to 22 years and pulmonary function measured at 22 years, females (n = 1620).

	Females– 22y		
	FEV_1_ (L) β (95% CI)	FVC (L) β (95% CI)	FEV_1_/FVC (%) β (95% CI)
**Fat mass (%) in the highest tertile at 18 and 22 years**	p< 0.001	p< 0.001	p = 0.049
No	Reference (0)	Reference (0)	Reference (0)
Only at 18	-0.016 (-0.064; 0.054)	-0.042 (-0.121; 0.038)	0.493 (-0.559; 1.544)
Only at 22	-0.175 (-0.248; -0.102)	-0.173 (-0.255; -0.090)	-0.760 (-1.861; 0.340)
Both ages	-0.192 (-0.260; -0.124)	-0.259 (-0.336; -0.182)	0.808 (-0.213; 1.829)
**FMI in the highest tertile at 18 and 22 years**	p< 0.001	p< 0.001	p = 0.031
No	Reference (0)	Reference (0)	Reference (0)
Only at 18	0.087 (0.014; 0.161)	0.105 (0.021; 0.188)	-0.126 (-1.224; 0.973)
Only at 22	-0.119 (-0.196; -0.042)	-0.080 (-0.167; 0.008)	-1.416 (-2.570; -0.263)
Both ages	-0.099 (-0.173; -0.025)	-0.130 (-0.215; -0.046)	0.227 (-0.888; 1.341)
**BMI ≥30 kg/m**^**2**^ **at 18 and 22 years**	p = 0.052	p = 0.008	p = 0.475
No	Reference (0)	Reference (0)	Reference (0)
Only at 18	-0.007 (-0.182; 0.168)	0.004 (-0.195; 0.202)	-0.388 (-3.003; 2.227)
Only at 22	-0.107 (-0.186; -0.028)	-0.137 (-0.227; -0.048)	0.200 (-0.981; 1.380)
Both ages	-0.108 (-0.210; -0.006)	-0.177 (-0.292; -0.061)	1.106 (-0.417; 2.629)

BMI: body mass index; FMI: fat mass index; FEV_1_: forced expiratory volume in the first second; FVC: forced vital capacity; β: regression coefficient, p-value by Wald’s test for heterogeneity. ADJUSTED for birth weight and maternal smoking in pregnancy, skin color, self-reported wheezing in the last year at 18 and 22 years, current smoking at 18 and 22 years, and height, weight, physical activity (minutes/week), use of corticosteroids in the last three months, education and asset index at 22 years follow-up.

The participants who moved from the highest tertile at 18 to the lowest FM or FMI tertiles or to BMI <30kg/m^2^ at 22 years, in the majority of the analyses, presented similar pulmonary function compared to the reference group. Also, FEV_1_ and FVC improved in those who migrated to the lower FMI categories at 22 years (Tables 3 and 4). Regarding FEV_1_/FVC ratio, most of the analyses were not significant, except higher values among man who kept higher FM in both ages (Table 3) and lower values among women who belonged to the higher FMI tertile only at 22 year (Table 4) when compared to the reference category.

## Discussion

The present study shows that high body adiposity at the end of the adolescence (18 years) and beginning of the adulthood (22 years) was associated with lower values of FEV_1_ and FVC at the onset of adulthood (22 years). In addition, changes in adiposity from 18 to 22 years of age, had an impact on lung function: decreasing adiposity category was associated to a lung function similar to or, in a few analyzes, higher than those who were in the reference categories in both evaluations, whereas changes to higher adiposity categories, mainly in females, were associated with lower mean values of FEV_1_ and FVC at 22 years. Reduced lung function, mainly FEV_1_, has been considered a predictor of adverse outcomes including overall, respiratory, cancer and cardiovascular mortality [25, 26].

S1 and S2 Tables show the same linear regression models adding the adjustment for previous lung function (the same parameter tested, measured at 18 year old). Most of the associations for being in the highest adiposity categories in both follow-ups were lost, probably due to previous lower pulmonary function already attributed to excess of fat [10]. On the other hand, most of the findings for those who changed adiposity categories from 18 to 22 years were reinforced.

Despite the limitations presented by BMI due to the limited distinction between fat mass and fat free mass, it is widely used and easily assessed [5, 9]. BMI was used in this study in order to compare its results using the cutoff point that defines obesity (30kg/m^2^) [9, 24] with those pointed out by FM and FMI. Previous studies have shown that the relationship between BMI and pulmonary function is not linear, due to the uncertainty between the percentage of FM and lean mass in the intermediate categories (normal/ overweight BMI), especially among adolescents and young adults [7, 10, 12, 13]. However, individuals with BMI ≥ 30kg/m^2^ have lower pulmonary function attributed to adiposity excess [7, 13].

Most of the longitudinal studies available on this subject use BMI as a measure of exposure and conclude that BMI gain or high BMI throughout life are associated with lower pulmonary function. Birth weight [27] and/or BMI from childhood [27–29] were not associated with pulmonary function at the onset of adulthood. Pulmonary function had an inverse association with BMI only among those with persistent overweight or in individuals with high BMI in adulthood [27–29]. These results are consistent with our findings, especially among women. Other longitudinal studies demonstrated a decline in pulmonary function with rises in BMI [30–33], even controlling for age and smoking.

In the cohort from 32 to 38 years of age [34], FM (bioelectrical impedance—BIA), BMI and waist circumference trajectories had inverse associations with pulmonary function measures. Fenger et al.[35] also obtained similar results using BIA and anthropometric measurements, but in a heterogeneous age group. We did not find any previous longitudinal study with the similar target age and precise methods for body composition as the present one.

This study also complements the 1993 Birth Cohort previous cross-sectional analysis. These analyses had shown a consistent inverse association between several adiposity measures and FEV_1_ and FVC at 18 years old, however, it was not possible to establish temporality because the measurements of body composition and pulmonary function were carried out at the same time [10]. In the present study we observed that males migrating to higher FM tertiles, or females to higher tertiles of FM or FMI or BMI ≥ 30kg/m^2^, showed lower values in the spirometric function than the reference, which demonstrates pulmonary function was related mainly with current adiposity in our cohort. Changes in lung function with increased adiposity category were easier to demonstrate in women, with any obesity marker maybe because of the higher proportion of body fat in females compared to males in this population. While the mean of FM in the highest tertile is 32.6% in men, women present 45.3% (data not presented in tables).

The FEV1 and FVC pattern through the analyses was similar, increasing or decreasing together. This demonstrates that the main mechanism of adiposity on lung function is probably due to restriction and not to obstruction. This also explains the lack of association between adiposity variables and FEV1/FVC ratio, as these parameters vary in similar magnitude, therefore the ratio remains relatively constant. It was observed that those subjects who migrated from the higher to the lower adiposity categories revealed similar or higher pulmonary function compared to the references in both sexes, showing that the deleterious effects of obesity on pulmonary function could be reversible. Pistelli et al. [30] found a similar result in a population of Italians, mean age 40 years. In addition, previously obese according to BMI, showed lower declines in FEV_1_ and FVC than those who were never obese in an 8 years interval between two follow-up [30]. Weight loss whether through hypocaloric diet [36, 37] or surgical interventions [38, 39], was also associated with improvements in pulmonary function tests. In our sample we observed that individuals who were in the highest FMI category only at 18 years, 81.3% among men and 60.2% among women reached the recommendation of at least 150min/week of exercises, percentage beyond the sample mean (Table 1). Participants were also asked about weight lost attempts through diet: 30.9% and 40.3%, among men and women who were in the highest FMI tertile only at 18 years, respectively, referred to have had a diet in the last 12 months before the 22 years follow-up.

This study has some limitations, which should be highlighted: a) we lack static lung volumes, often used in the literature to evaluate pulmonary function in relation to body composition [6, 11, 16, 40] but on the other hand, spirometry is the most reliable test evaluating mechanics of breathing; and b) although anthropometric measures were taken since infancy, we do not have BOD POD before 18 years of age and were unable to assess precise measurement of body composition throughout life.

As strengths, our large birth cohort, representative of the population and with little risk of selection bias, has high follow-up rates and includes information for several confounding factors, allowing statistical analyses with high power. Spirometry tests had a high quality and we used high precision devices to evaluate the body composition, uncommon among population-based studies. The 1993 Pelotas Birth Cohort is planned to continue the follow-ups of its participants through their adult age, allowing future analyzes on this subject.

## Conclusion

Lung function in those reducing adiposity categories from high to lower tertiles at 18 and 22 years of age, was similar to those always in the lower tertiles. High adiposity in both evaluations was associated with lower FEV_1_ and FVC; similar to what happened in those who migrated to higher adiposity categories at age 22, although in lower magnitude, especially among women. These adverse impacts of obesity on lung function in young individuals are worrisome and have potential long-term consequences.

## Supporting information

S1 File Analysis dataset (Stata v. 12). (DTA)

S1 Table Adjusted linear regressions between body adiposity from 18 to 22 years and pulmonary function measured at 22 years, adding adjustment for previous pulmonary function, males (n = 1494). (DOCX)

S2 Table Adjusted linear regressions between body adiposity from 18 to 22 years and pulmonary function measured at 22 years, adding adjustment for previous pulmonary function, females (n = 1620). (DOCX)
