# Supplementary material for: Body composition from 18 to 22 years and pulmonary function at 22 years—1993 Pelotas Birth Cohort
Source: PLoS One. 2019 Jun 27;14(6):e0219077. doi: 10.1371/journal.pone.0219077 (PMC6597105; doi:10.1371/journal.pone.0219077)
Supplement: S1 Table — (DOCX) [file pone.0219077.s002.docx]

Supplementary Table 1. Adjusted linear regressions between body adiposity from 18 to 22 years and pulmonary function measured at 22 years, adding adjustment for previous pulmonary function, males (n = 1494).

|  | **Males - 22y** | | |
| --- | --- | --- | --- |
|  | **FEV_1_ (L)**  **β (95% CI)** | **FVC (L)**  **β (95% CI)** | **FEV_1_/FVC (%)**  **β (95% CI)** |
| **Fat mass (%) in the highest tertile at 18 and 22 years** | p= 0.002 | p= 0.043 | p= 0.019 |
| No | Reference (0) | Reference (0) | Reference (0) |
| Only at 18 | 0.062 (0.007; 0.116) | 0.009 (-0.043; 0.060) | 1.053 (0.289; 1.818) |
| Only at 22 | -0.083 (-0.144; -0.022) | -0.074 (-0.133; -0.016) | -0.244 (-0.099; 2.531) |
| Both ages | -0.032 (-0.087; 0.023) | -0.049 (-0.101; 0.004) | 0.566 (-0.196; 1.328) |
| **FMI in the highest tertile at 18 and 22 years** | p= 0.003 | p= 0.076 | p= 0.007 |
| No | Reference (0) | Reference (0) | Reference (0) |
| Only at 18 | 0.084 (0.026; 0.142) | 0.021 (-0.034; 0.076) | 1.219 (0.403; 2.439) |
| Only at 22 | -0.059 (-0.122; 0.004) | -0.056 (-0.116; 0.004) | -0.141 (-1.030; 0.749) |
| Both ages | -0.019 (-0.077; 0.039) | -0.054 (-0.108; 0.001) | 0.802 (-0.003; 1.607) |
| **BMI ≥30 kg/m² at 18 and 22 years** | p= 0.002 | p= 0.017 | p= 0.006 |
| No | Reference (0) | Reference (0) | Reference (0) |
| Only at 18 | 0.184 (0.062; 0.306) | 0.133 (0.018; 0.249) | 1.389 (-0.322; 3.100) |
| Only at 22 | -0.008 (-0.079; 0.063) | -0.062 (-0.129; 0.006) | 0.839 (-0.162; 1.840) |
| Both ages | -0.105 (0.015; 0.194) | -0.007 (-0.078; 0.092) | 2.109 (0.860; 3.356) |
| BMI: body mass index; FMI: fat mass index; FEV_1_: forced expiratory volume in the first second; FVC: forced vital capacity; β: regression coefficient, p-value by Wald’s test for heterogeneity.  ADJUSTED for birth weight and maternal smoking in pregnancy, skin color, self-reported wheezing in the last year at 18 and 22 years, current smoking at 18 and 22 years, and height, weight, physical activity (minutes/week), use of corticosteroids in the last three months, education, asset index at 22 years follow-up AND FEV_1_, FVC or FEV_1_/FVC at 18 years follow-up. | | | |
