# Supplementary material for: Body composition from 18 to 22 years and pulmonary function at 22 years—1993 Pelotas Birth Cohort
Source: PLoS One. 2019 Jun 27;14(6):e0219077. doi: 10.1371/journal.pone.0219077 (PMC6597105; doi:10.1371/journal.pone.0219077)
Supplement: S2 Table — (DOCX) [file pone.0219077.s003.docx]

Supplementary Table 2. Adjusted linear regressions between body adiposity from 18 to 22 years and pulmonary function measured at 22 years, adding adjustment for previous pulmonary function, females (n = 1620).

|  | **Females – 22y** | | |
| --- | --- | --- | --- |
|  | **FEV_1_ (L)**  **β (95% CI)** | **FVC (L)**  **β (95% CI)** | **FEV_1_/FVC (%)**  **β (95% CI)** |
| **Fat mass (%) in the highest tertile at 18 and 22 years** | p< 0.001 | p= 0.046 | p< 0.001 |
| No | Reference (0) | Reference (0) | Reference (0) |
| Only at 18 | 0.018 (-0.023; 0.060) | -0.015 (-0.058; 0.027) | 0.886 (0.156; 1.615) |
| Only at 22 | -0.084 (-0.127; -0.041) | -0.026 (-0.071; 0.018) | -1.451 (-2.210; -0.691) |
| Both ages | -0.055 (-0.095; -0.014) | -0.060 (-0.101; -0.018) | 0.208 (-0.498; 0.914) |
| **FMI in the highest tertile at 18 and 22 years** | p= 0.002 | p= 0.380 | p< 0.001 |
| No | Reference (0) | Reference (0) | Reference (0) |
| Only at 18 | 0.034 (-0.009; 0.078) | -0.002 (-0.046; 0.043) | 0.791 (0.027; 1.555) |
| Only at 22 | -0.074 (-0.119; -0.029) | -0.020 (-0.066; 0.026) | -1.400 (-2.197; -0.604) |
| Both ages | -0.026 (-0.070; 0.018) | -0.039 (-0.084; 0.006) | 0.324 (-0.447; 1.096) |
| **BMI ≥30 kg/m² at 18 and 22 years** | p= 0.076 | p= 0.007 | p= 0.234 |
| No | Reference (0) | Reference (0) | Reference (0) |
| Only at 18 | 0.034 (-0.067; 0.135) | -0.030 (-0.132; 0.073) | 1.328 (-0.455; 3.112) |
| Only at 22 | -0.060 (-0.108; -0.012) | -0.083 (-0.131; -0.034) | 0.291 (-0.556; 1.138) |
| Both ages | -0.037 (-0.097; 0.023) | -0.083 (-0.144; -0.022) | 0.869 (-0.196; 1.934) |
| BMI: body mass index; FMI: fat mass index; FEV_1_: forced expiratory volume in the first second; FVC: forced vital capacity; β: regression coefficient, p-value by Wald’s test for heterogeneity.  ADJUSTED for birth weight and maternal smoking in pregnancy, skin color, self-reported wheezing in the last year at 18 and 22 years, current smoking at 18 and 22 years, and height, weight, physical activity (minutes/week), use of corticosteroids in the last three months, education, asset index at 22 years follow-up AND FEV_1_, FVC or FEV_1_/FVC at 18 years follow-up. | | | |
